# Steatotic liver disease among lean and non-lean individuals in Southern Lao PDR: a cross-sectional study of risk factors

**DOI:** 10.1080/07853890.2024.2329133

**Published:** 2024-03-19

**Authors:** Anousin Homsana, Phonesavanh Southisavath, Kerstin Kling, Jan Hattendorf, Savina Vorasane, Daniel Henry Paris, Somphou Sayasone, Peter Odermatt, Nicole Probst-Hensch

**Affiliations:** a Lao Tropical and Public Health Institute, Ministry of Health, Vientiane Capital, Lao PDR; b Swiss Tropical and Public Health Institute, Allschwil, Switzerland; c University of Basel, Basel, Switzerland; d Department of Radiology, Mahosot Hospital, Ministry of Health, Vientiane Capital, Lao PDR; e Immunization Unit, Robert Koch Institute, Berlin, Germany

**Keywords:** Lao PDR, steatotic liver disease, hepatic steatosis, metabolic dysfunction-associated steatotic liver disease, type 2 diabetes mellitus, metabolic diseases, *opisthorchis viverrini*, lipids

## Abstract

**Background:**

Steatotic liver disease (SLD) prevalence is rising worldwide, linked to insulin resistance and obesity. SLD prevalence can surpass 10% even among those with normal weight. In Lao People’s Democratic Republic (Lao PDR), where *Opisthorchis viverrini* (OV) trematode infection and type 2 diabetes mellitus (T2DM) are common, infection related liver morbidity such as cholangiocarcinoma (CCA) is high, but data on SLD prevalence is lacking. The objective of this study was to estimate the prevalence and explore determinants of SLD in rural southern Lao PDR for lean and non-lean populations.

**Method:**

A cross-sectional community-based study assessed SLD prevalence using abdominal ultrasonography (US). Factors investigated for association with SLD were identified by interview, serological tests (Hepatitis B surface antigen (HBsAg); lipids and HbA1c), anthropometrical measurements, and parasitological assessments (OV infection). Uni- and multivariable logistic regression analyses with SLD as endpoint were conducted separately for lean (body mass index (BMI) <23.0 kg/m^2^) and non-lean (BMI ≥ 23.0 kg/m^2^) participants.

**Result:**

2,826 participants were included. SLD prevalence was 27.1% (95% confidence interval (95% CI) 24.0%−30.4%), higher among non-lean (39.8%) than lean individuals (17.4%). Lean individuals with OV infection had a statistically significant association with lower odds of SLD (adjusted odds ratio (aOR) 0.49, 95% CI 0.33 − 0.73). T2DM showed a significant positive association with SLD in both lean (aOR 3.58, 95% CI 2.28 − 5.63) and non-lean individuals (aOR 3.31, 95% CI 2.31 − 4.74) while dyslipidemia was significantly associated only in the non-lean group (aOR 1.83, 95% CI 1.09 − 3.07). Females participants exhibited elevated odds of SLD in both lean (aOR 1.43, 95% CI 1.02 − 2.01) and non-lean SLD (aOR 1.50, 95% CI 1.12 − 2.01).

**Conclusion:**

SLD prevalence is notably high among Laotian adults in rural areas, particularly in females and in non-lean individuals. Lean individuals with OV infection exhibited lower SLD prevalence. SLD was more prevalent in individuals with T2DM, independent of BMI. SLD adds to the burden of infection-related liver morbidity in Lao PDR.

## Introduction

Steatotic liver disease (SLD, previously known as fatty liver disease or FLD) is a condition characterized by the accumulation of excess fatty particles (triglycerides) in the liver parenchyma [1] and is thought to be a response to a wide range of hepatic stressors and toxins [2]. SLD includes metabolic alcoholic liver disease (MetALD) and metabolic dysfunction-associated steatotic liver disease (MASLD, formerly known as non-alcoholic fatty liver disease or NAFLD). MASLD is the most common SLD subclass globally and not primarily related to excessive alcohol consumption or infections (e.g. with hepatitis B virus (HBV) or hepatitis C virus (HCV)) [3].

SLD prevalence has increased worldwide in recent decades, in line with the rise in key contributing factors such as metabolic syndrome (MetS)-related phenotypes, alcohol consumption, as well as demographic aging of populations [1,4,5]. The relationship between SLD and MetS is bidirectional. SLD can progress to more serious chronic liver diseases, such as metabolic dysfunction-associated steatohepatitis (MASH, formerly known as non-alcoholic steatohepatitis or NASH), irreversible liver fibrosis, and hepatocellular carcinoma (HCC). Less than 10% of SLD patients progress within 10-20 years of diagnosis to HCC, but among them, mortality from liver disease is high [3,6]. SLD is a major predisposition for liver transplants in high-income countries.

SLD is the most common chronic liver condition, with a global prevalence of about 25%, varying from 14% in African settings to 32% in the Middle East [6,7]. In Asian countries, a meta-analysis indicated a prevalence of 30% for SLD between 1999 and 2019, with an increasing trend (almost 34% between 2012 and 2017) [5]. Although obesity and metabolic syndrome-related factors are present in far more than 50% of SLD patients and play a key etiologic role [1,6,8,9], between 10% and 20% of individuals living in Asia with body mass indices (BMI) below 23 kg/m^2^ [10] are found to have ‘lean’ SLD. These lean SLD patients may present in part with central adiposity [6] as well as possibly pancreatic *β*-cell dysfunction [9]. Understanding of ‘lean’ SLD remains limited [11].

The interactions between metabolic factors, chronic infections, and excess alcohol intake in the etiology of ‘lean’ and ‘non-lean’ SLD are understudied, despite their common co-occurrence in many low- and middle-income settings.

The low income country (LIC) of Lao People’s Democratic Republic (Lao PDR) carries a high burden of chronic liver morbidity as a result of the estimated 2 million citizens (about 30% of total population) infected with the trematode *Opisthorchis viverrini* (OV) [12], a major cause of cholangiocarcinoma (CCA). Simultaneously, a high prevalence of prediabetes (37%) and diabetes (23%) determined by HbA1c levels was observed in adults aged 35 years and older in Lao PDR [13]. Data on the SLD prevalence in Lao PDR is nevertheless sparse and has so far only been reported in two studies using abdominal ultrasound (US). The first study found a prevalence of 12% among participants aged above 20 years in southern Lao PDR [14], while the second (participants spanned a broader range of ages of below 20 years and exceeding 80 years) reported SLD prevalence of 11% [15]. These estimates are below those observed in the neighboring country of Thailand, where SLD prevalence ranged from 21% to 50% [16,17].

This cross-sectional study aimed to estimate the prevalence and socio-demographic distribution of abdominal ultrasound-derived SLD in rural areas of southern Lao PDR with a high OV prevalence. It also investigated the association of lifestyle, infections, and metabolic syndrome with both ‘lean’ SLD and ‘non-lean’ SLD.

## Materials and methods

### Ethical considerations

The study received approval by the National Ethics Committee for Health Research, Ministry of Health (MoH), Vientiane, Lao PDR (Ref. no. 113/2018 NECHR) and the responsible Swiss ethics committee (Ethikkommission der Nordwest- und Zentralschweiz; EKNZ, Ref. no. R-2017-00869). Permission for the fieldwork was obtained from the MoH, the Provincial Health Offices of Champasak (CPS) and Savannakhet (SVK), the District Health Offices, and the District Office of Education and Sports of Champhone and Khong districts.

Before commencing data collection, meetings were conducted in each village. Village authorities and residents received comprehensive explanations regarding study’s objectives, procedures, potential risks, and benefits. A consent form was read aloud in Lao to the participants, and their questions were addressed. Participants provided written consent prior to their enrollment. Consent forms were stored separately from the research data, ensuring privacy.

Participants had the option to be informed about the results of their US and parasitological examinations. Those diagnosed with SLD received advice from the study doctors on physical exercise, and healthy diets. Participants infected with OV were treated with Praziquantel (PZQ) according to the Lao national treatment guidelines [18]. If participants were suspected of having CCA, they were contacted for further radiological follow-up. Once CCA was confirmed, the surgical operation was performed at Mahosot hospital in Vientiane, the capital of Lao PDR. All treatments and travel costs to Vientiane were provided to the participants free of charge.

## Study design, sample size, sampling, eligibility criteria, and fieldwork procedures

This cross-sectional study was integrated into a larger study with the primary objective of identifying risk factors for suspected CCA. The sample size calculation was based on the association between key risk factors and suspected CCA, considering the proportion needed to detect a difference between advanced periductal fibrosis (APF+) and absent periductal fibrosis (APF-) patients [19]. Assuming a ratio of 1:2 for persons exposed to a certain risk factor compared to non-exposed individuals and the proportion of approximately 15% APF + participants as indicated in prior work [14], a sample size of 2,100 participants allows detecting a difference of 5% points with 80% power at a 95% confidence level. The sample size calculations were performed for the primary objective as outlined above, whereas in this study SLD is the endpoint of interest, and individuals with suspected CCA and liver cirrhosis were excluded.

This study took place in Khong (CPS province) and Champhone (SVK province) districts between December 2017 and February 2019. In these provinces, OV infection is highly endemic due to raw-fish ingestion and the presence of large fresh water bodies (e.g. Mekong River). In 2015, the total population size of Champasak and Savannakhet was 970,000 and 694,000 people, respectively (http://www.nsc.gov.la). Twenty-one study villages (12 in CPS and 9 in SVK) were selected in collaboration with the district health offices, considering population size and logistic feasibility. Among the total of 6,450 eligible persons (aged ≥35 years old) listed in the resident registration, encompassing all villages, 3,879 individuals indicated their interest by providing consent and submitting 1^st^ initial fecal sample. Out of these, 3,583 participants took part in the US examination, resulting in an average participation rate of 55.6% for the US component (refer to Appendix 1).

The eligibility criteria for participation included: (i) residents from each village, and (ii) individuals aged 35 years and older. Exclusion criteria encompassed: (i) pregnancy (due to challenges in performing abdominal ultrasound examinations in this group), and (ii) residents who had recently moved into the villages (i.e. within the past 5 years given the long latency of cancer occurrence and that participants from other areas might not share the same risk of OV infection compared to residents in OV endemic areas), (iii) individuals with liver cirrhosis and/or suspected cholangiocarcinoma (to ensure clear associations between risk factors and SLD without influence of pre-existing liver abnormalities).

Each interested participant was required to attend the examination, which ranged from 3-5 days. The process was divided into three sequential days: (i) on the first day, consenting participants were registered and provided plastic containers labeled with participant IDs for stool collection. (ii) On the second day, participants brought their filled fecal containers to the designated study point in the village for processing and examination, which took place on the same day. Additionally, face-to-face interviews were conducted. Participants were also given a second stool collection container along with instructions to fast in preparation for the third day’s examination. (iii) On the third and final day, participants arrived in the morning to undergo all remaining examinations, including abdominal ultrasound, physical examination, and blood drawing. The second fecal sample was also processed on this same day.

Throughout the data collection process, 25-50 participants were recruited daily, with the recruitment of new participants and examination of existing participants occurring concurrently each day until all voluntary participants in the village had been included. The duration of data collection in each village varied between 1 to 3 weeks, depending on the number of volunteering study participants.

A 5 ml venous blood sample was collected from each participant. The drawn whole blood samples were stored in EDTA containers, and serum was obtained by centrifugation at 1,500 rpm for 10 min at room temperature. All blood aliquots were immediately stored at −20 °C at the study sites for a maximum period of 4 weeks and thereafter transported to the laboratory at the Lao Tropical and Public Health Institute (Lao TPHI) in Vientiane. The cold chain was maintained using portable mobile sample freezers designed for vehicle use. At Lao TPHI serum and ETDA blood were stored at −80 °C until testing.

### Research tools and measurements

#### Abdominal ultrasonography

Liver morbidity was assessed using a mobile US device (Mindray Z6, Shenzhen Mindray Bio-Medical Electronics, Shenzhen, China), equipped with convex (model 3C5P) and linear (model 7L4P) probes in B-mode (2-dimensional image mode).

**Figure 1.**
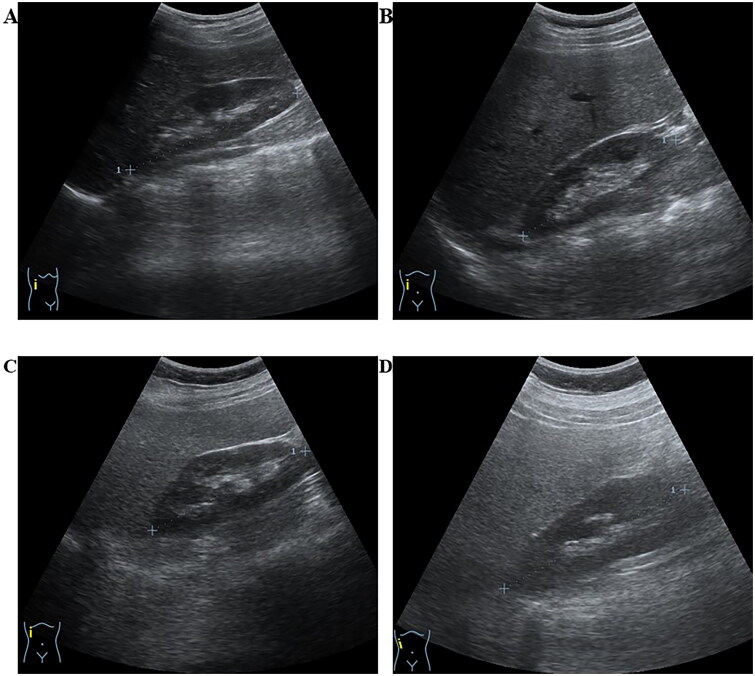
Ultrasound imaging depicting stages of steatotic liver disease (SLD). The figure presents ultrasound images illustrating steatotic liver conditions in four different participants in this study. Liver steatosis was assessed by qualitatively comparing the echogenicity of the liver parenchyma to the echogenicity of the right kidney [20]. (A) Normal liver, (B) Mild liver steatosis, (C) Moderate liver steatosis, and (D) Severe liver steatosis. The grading criteria for fatty liver included diaphragm images, which are not depicted in this figure.

SLD was categorized into three grades based on the increased echogenicity of the liver parenchyma compared to right kidney, portal veins, and diaphragm (Figure 1). The classification include: (i) mild ─ indicating a slight increase in liver echogenicity, (ii) moderate ─ suggesting slightly impaired intrahepatic vessels and diaphragm with increased echogenicity, and (iii) severe ─ characterized by a marked increase in intrahepatic echogenicity with poor penetration to the posterior segment of the right liver lobe and poor or no visualization of hepatic vessels and diaphragm [20].

#### Questionnaire

Information on socio-demographic characteristics (individuals aged 35 years and above), household assets, and livestock (such as fish pond, chicken, cow, pig, water buffalo, rice cooker, radio, fan, refrigerator, television, bicycle, motorbike, farm vehicle, place to grow vegetables, cement floor, and tap water) was obtained through questionnaire-based in-person interviews. The questionnaire on lifestyle-related risk factors covered various aspects. Participants were queried about their smoking habits (current, previous, or non-smoker). Consumption of any alcoholic beverage was assessed by inquiring about the frequency of drinking (non-drinkers, monthly, 1-2 times/week, 3-6 times/week, and ≥ once per day). Dietary behaviors, such as the consumption of raw fish, were documented. The intake frequencies of sweet beverages (e.g. soft drinks and sugary drinks), sweet & salty snacks (e.g. salty potato chips, sweet chocolate, etc.), and fruits and vegetables were recorded in days per week. Medical history information included self-reported diagnoses or medication intake for type 2 diabetes mellitus (T2DM), hypertension, or dyslipidemia. The interviews typically lasted between 15 and 20 min.

#### Opisthorchis viverrini infection

Fecal samples were examined using the Kato-Katz technique [21]. Two Kato-Katz smears per sample were established and evaluated (total 4 smears). The smears were prepared according to the producer’s instructions and allowed to clear for 30 to 60 min before examination under light microscopes by experienced microscopists. All OV and other helminth eggs were counted by each species separately.

#### HbA1c, lipid profiles and hepatitis B status

All laboratory examinations were conducted at Lao TPHI, e.g. assessment of HbA1c, lipid profiles, and hepatitis B status. The measurement of hemoglobin A1c (HbA1c %) levels was performed using EDTA blood samples. For lipid profile analysis, serum samples were utilized to measure high-density lipoprotein (HDL) (mg/dL), low-density lipoprotein (LDL) (mg/dL), triglyceride (TG) (mg/dL), and total cholesterol (TC) (mg/dL) levels. Both HbA1c and lipid profile measurements were conducted using an enzymatic assay method on an automated commercially available clinical chemistry analyzer (Mindray model: BS-240, Mindray Corporation, Shenzhen, China), following the manufacturer’s instructions. The Mindray BS-240 enzymatic method has been certified by NGSP for laboratory testing of HbA1C (HbA1C ≥6.5% indicates diabetes diagnosis) [22,23]. The testing process employed ready-to-use reagents, calibrators, and control sets for each parameter, all of which were sourced from the same manufacturer as the analyzer. Serum samples were also utilized to analyze HBV infection using the HBV rapid diagnostic test (Vikia HbsAg, bioMérieux, France).

#### Anthropometry

Weight was measured to the nearest 0.1 kg (Seca, model: M 877, Hammer Steindamm 3-2522089 Hamburg, Germany). Height was measured without shoes to the nearest 0.5 cm (Seca, model: 206, Hammer Steindamm 3-2522089 Hamburg, Germany). BMI was calculated by the formula weight in kilograms divided by height in meters squared. Participants were classified into lean (BMI < 23.0 kg/m^2^) and non-lean (BMI ≥ 23.0 kg/m^2^) groups using the recommended body-mass index for Asian populations [11].

#### Blood pressure

Measurements were taken from both mid-upper arms of participants. The measurements were conducted at least 5 min apart between both arms and performed after participants had been in the resting or seated position for at least 5 min using an automatic blood pressure monitor (Omron HEM-8712, Omron, Hoofdoorp, Netherlands).

### Data management and statistical analysis

Data was recorded using tablets preinstalled with Commcare ODK mobile database allowing for direct data upload to Commcare server (www.commcarehq.org, version 3.4). Data analysis was performed using STATA software, version 16.0 (StataCorp, College Stata, TX, USA). Figures were created in Stata and R (v 4.0.2).

In a first step, the overall prevalence rate of SLD, along with its corresponding 95% confidence interval (95% CI), was derived using logistic regression constant-only random-effect model. The distribution of overall SLD was described as frequency and proportion according to socio-economic characteristics (sex, age (35-49 years; 50-59 years; ≥60 years), education, profession, province, and socio-economic status (SES)). SES was determined by the component scores of household assets and livestock ownership, calculated using principle factor analysis, and categorized into tertile labeled poor, middle, and wealthy [24]).

In a second step, the distribution of BMI scores was presented using boxplots according to the presence and severity stages of SLD.

In a third step, the distribution of socio-demographic characteristics, lifestyle factors and health indicators was described as frequency and proportion according to lean SLD and non-lean SLD. Alcohol consumption was categorized as daily vs. less frequent; smoking status as never, former and current; hypertension as systolic BP ≥ 140 mmHg and/or diastolic BP≥ 90 mmHg and/or a self-reported diagnosis of hypertension and/or a self-reported intake of blood pressure lowering medication [25]; and T2DM as one of HbA1c ≥ 6.5% and/or self-reported diagnosis of T2DM and/or self-reported intake of T2DM medication. Dyslipidemia was defined as TC ≥ 200mg/dL and/or TG ≥ 150mg/dL and/or LDL ≥ 150mg/dL and/or HDL ≤ 40mg/dL and/or self-report of medical treatment for high blood lipids. A healthy diet index was created, where daily consumption of fruits, daily consumption of vegetables, non-daily consumption of sweet beverages, non-daily consumption of sweet snacks, and non-daily consumption of salty snacks each contributed 1 point to the healthy diet index. A higher healthy diet index reflected a healthier diet. A positive OV infection status was determined by the presence of OV eggs in any microscopic slides. A positive HBV status was defined as a positive HBV’s surface antigens (HBsAg).

In a fourth step, the associations of lifestyle factors and health indicators, adjusted for socio-economic and -demographic variables, with binary SLD as a dependent variable were assessed using univariate and multivariable random-effects logistic regression models (with village as random effect to account for potential correlations within villages). Blood pressure was excluded from the multivariable model, because it can be both a mediator from obesity and T2DM to SLD as well as an outcome of SLD. The models were run separately for participants with BMI < 23.0 kg/m^2^ and ≥23.0 kg/m^2^. Sensitivity analysis was performed to assess the robustness of the fully adjusted models. These analyses examined the influences of alcohol consumption and HBV infection, as well as different BMI cutoff points for lean and non-lean groups (<25.0 kg/m^2^ and ≥25.0 kg/m^2^). Crude odds ratio (cOR), adjusted odds ratio (aOR) and the corresponding 95% CI were reported for the results of the logistic regression model.

## Results

**Figure 2.**
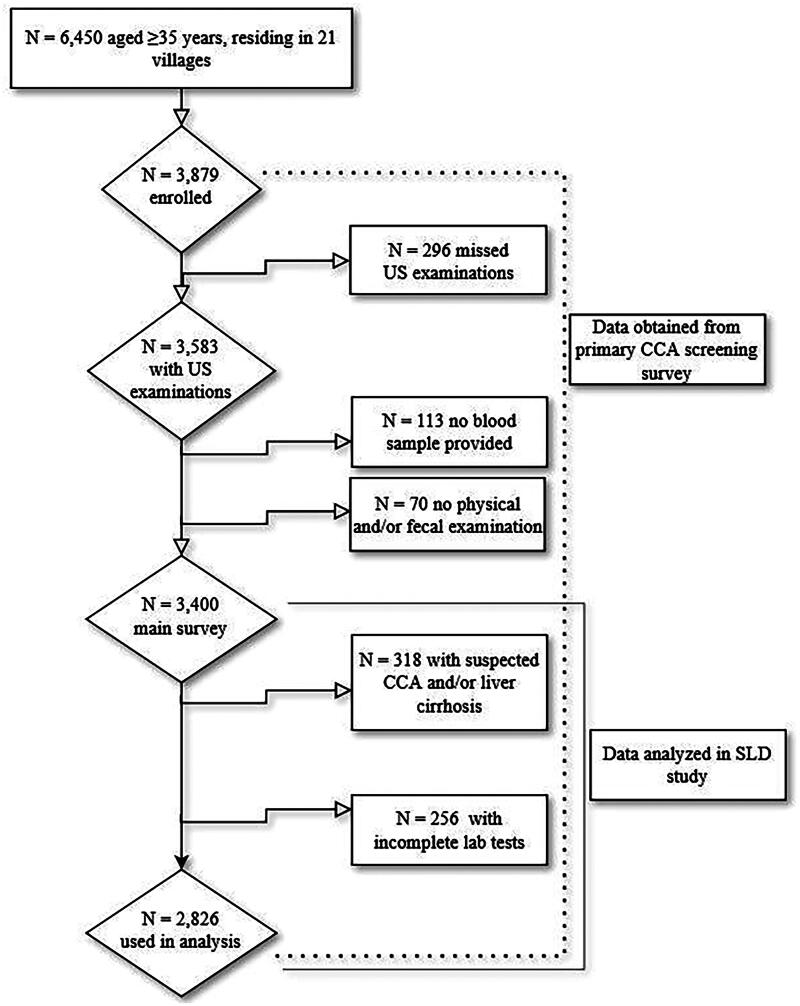
Study diagram.

Out of 6,450 residents aged 35 and older listed in the village registration books, 3,583 completed the US examination, resulting in an average participation rate of 55.6% (refer to Appendix 1). Out of the 3,583 participants, 2,826 participants were available for analysis. The reasons for missing data were as follows: several individuals missed one of the examination activities, e.g. blood draw, physical and parasitology examinations (*n* = 180); had incomplete laboratory testing (*n* = 256); or showed other liver morbidities, including suspected CCA and/or liver cirrhosis (*n* = 318) (Figure 2).

### Socio-demographic characteristics of the study participants

The majority of participants (53.8%) were between 35 and 49 years old. The proportion of women was higher than men (60.9% vs. 39.1%). Approximately half of the participants (47.0%) had completed a primary school education, and the majority (78.7%) were farmers and/or laborers. Half of the participants originated from CPS, and the other half from SVK province. For a comprehensive overview of socio-demographic factors, see Table 1.

**Table 1.** Distribution of socio-demographic and economic factors among study participants, overall and according to the absence or presence of steatotic liver disease.

	Characteristics	All subjects (*n* = 2,826)	By any SLD, n (%)	
		N (Column %)	Non SLD	Any SLD
Overall	–	–	2,049 (72.5)	777 (27.1%, 95% CI 24.0%−30.0%)
Sex	male	1,104 (39.1)	856 (77.5)	248 (22.5)
	female	1,722 (60.9)	1,193 (69.3)	529 (30.7)
Age	35-49yr	1,520 (53.8)	1,112 (73.2)	408 (26.8)
	50-59yr	757 (26.8)	519 (68.6)	238 (31.4)
	≥60yr	549 (19.4)	418 (76.1)	131 (23.9)
Education	illiterate	482 (17.1)	347 (72.0)	135 (28.0)
	primary school	1,329 (47.0)	945 (71.1)	384 (28.9)
	above secondary school	1,015 (35.9)	757 (74.6)	258 (25.4)
Profession	housewife/elderly	246 (8.7)	179 (72.8)	67 (27.2)
	farmer/labour	2,223 (78.7)	1,638 (73.7)	585 (26.3)
	civil servants or traders	357 (12.6)	232 (65.0)	125 (35.0)
Socio-economic status	poor tertile	1,049 (37.1)	789 (75.2)	260 (24.8)
	middle tertile	911 (32.2)	673 (73.9)	238 (26.1)
	wealthy tertile	866 (30.6)	587 (67.8)	279 (32.2)
Province	CPS	1,395 (49.4)	947 (67.9)	448 (32.1)
	SVK	1,431 (50.6)	1,102 (77.0)	329 (23.0)

SLD - steatotic liver disease; yr - year; SES - socio-economic status; CPS - Champasak; SVK - Savannakhet, 95% CI - 95% confidence interval.

Data is presented in terms of frequency and row percentage, except for the overall column (column percent).

### Distribution of steatotic liver disease

The observed prevalence of SLD was 27.1% (95% CI 24.0%−30.0%). Stratified by severity categories, the prevalence was 16.4% mild, 9.9% moderate, and 1.2% severe SLD. SLD was more prevalent in females (30.7% vs. 22.5% in males) and in individuals between 50-59 years (31.4% vs. 26.8% in the 35-49 group and 23.9% in the ≥60 group). Furthermore, SLD was more prevalent among civil servants/traders group (35.0% vs. 27.2% in housewife/elderly group and 26.3% in farmers/labour group), individuals in the wealthy SES tertile (32.2% vs. less than 26.1% in other groups), and individuals living in CPS province (32.1% vs. 23.0% in SVK province) (Table 1).

**Figure 3.**
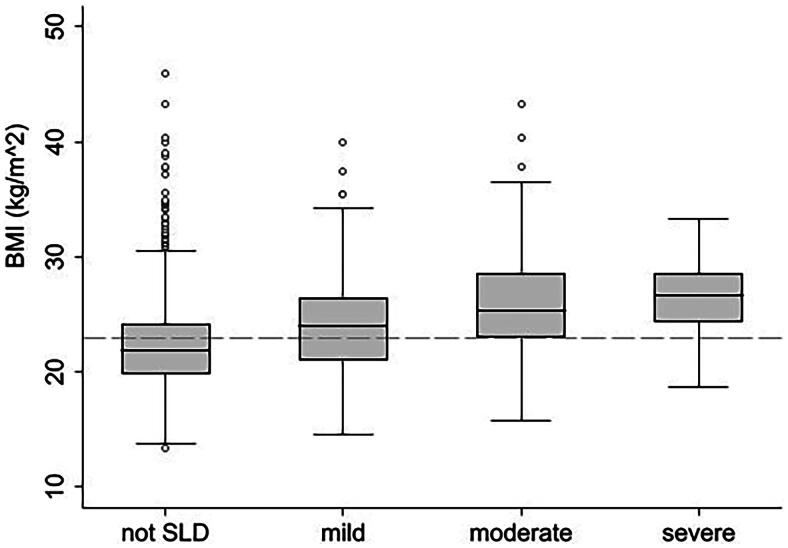
Distribution of BMI levels according to steatotic liver disease severity. Note: dash line on y-axis represents a BMI level of 23.0 kg/m^2^; SLD - steatotic liver disease.

Mean BMI was higher in persons with SLD compared to persons without SLD and increased with SLD severity level (Figure 3).

**Figure 4.**
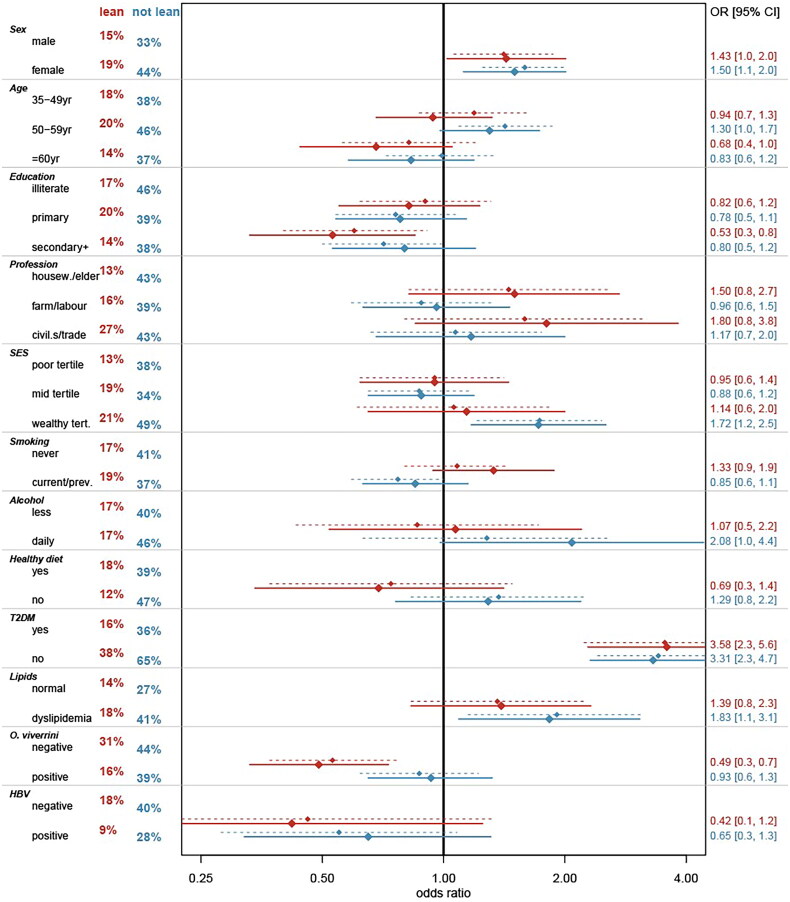
Bivariate and multivariate associations between covariates and fatty liver disease, stratified by body mass index (lean < 23.0 kg/m^2^ and non-lean ≥23.0 kg/m^2^). Notes: SLD - steatotic liver disease; yr - year; SES - social-economic status; T2DM - type 2 diabetes mellitus; OV - *Opisthorchis viverrini*; HBV - hepatitis B virus; Red color - indicates lean; blue color: indicates non-lean; percentages on the left side: proportion of steatotic liver; dotted lines: bivariate model; solid lines: multivariate model; OR [95% CI]: odds ratio [95% confidence intervals]. Percentages in red and blue on the left represent the prevalence of steatotic liver disease.

The prevalence of SLD in non-lean individuals was more than twice that in the lean group (39.8% vs. 17.4%) (Figure 4; Appendix 2). Descriptively, the distribution of SLD in subgroups of socio-economic factors was similar among lean and non-lean persons, except for the fact that in lean persons the difference in SLD prevalence between CPS and SVK (24.9% vs. 8.8%) was considerably larger than among non-lean persons (42.5% vs. 37.6%). No material differences in the prevalence of either lean or non-lean SLD were observed between subgroups of smoking or levels of healthy diet, or among persons consuming alcohol daily. SLD was particularly more prevalent in individuals with T2DM (lean: 38.4% vs. 15.8%; non-lean: 65.3% vs. 36.0%), increased blood pressure (lean: 21.7% vs. 16.5%; non-lean: 46.7% vs. 37.2%), and dyslipidemia (lean: 17.8% vs. 13.7%; non-lean: 40.7% vs. 27.1%). The prevalence of SLD was generally higher among participants without OV infection, with the difference being more pronounced among lean persons (lean: 30.6% vs. 15.6%; non-lean: 43.5% vs. 39.2%).

### Risk factors for fatty liver disease

Confirming the descriptive distribution of SLD among lean and non-lean individuals, the multivariate model revealed associations with various risk factors. Noteworthy, the associations of SLD with these risk factors did not differ in a statistically significant manner between lean and non-lean persons for most variables. An exception was OV infection status (Figure 3 and Appendix 3). In lean individuals, a positive OV status was associated with decreased odds of having SLD (aOR 0.49, 95% CI 0.33 − 0.73), whereas no statistical significant association was observed among non-lean participants infected with OV. Positive HBV status also showed a tendency of decreased odds for SLD, independent of BMI, although it did not reach statistical significance. In both lean and non-lean individuals, the presence of T2DM was associated with increased odds of SLD (aOR_lean_ 3.58, 95% CI 2.28 − 5.63; aOR_non-lean_ 3.31, 95% CI 2.31 − 4.74). In the non-lean group, the presence of dyslipidemia (aOR 1.83, 95% CI 1.09 − 3.07), daily alcohol intake (aOR 2.08, 95% CI 0.98 − 4.42), and being in the wealthiest tertile (aOR 1.72, 95% CI 1.17 − 2.53) were associated with increased odds of having SLD; however these associations did not reach statistical significance in the lean groups. Additionally, the odds of having SLD, whether lean or non-lean SLD, were higher in females compared to males (aOR_lean_ 1.43; 95% CI 1.02 − 2.01; aOR_non-lean_ 1.50, 95% CI 1.12 − 2.01).

In sensitivity analysis (refer to Appendix 4), no substantial differences were observed in the covariates associated with both, lean and non-lean SLD.

## Discussion

This first report on SLD in a rural Lao PDR setting, where both OV infections and diabetes are common, indicates a higher overall SLD prevalence of 27.1% (95% CI 24.0%−30.0%) compared to previous surveys. It was more than two-times higher in non-lean participants (39.8%), but even reached 17.4% in lean individuals. The presence of OV infections was inversely associated with lean SLD. T2DM was positively associated with both, non-lean and lean SLD, whereas dyslipidemia and daily alcohol consumption were positively associated with non-lean SLD only. Women exhibited higher odds of both lean and non-lean SLD even after adjusting for metabolic, sociodemographic, lifestyle and infection related covariates.

Compared to other countries, the observed prevalence of SLD in rural Lao PDR was somewhat higher than the 22% reported for the northeastern part of Thailand [16], but lower than the pooled estimate of 30% for MASLD in Asia as a whole [5]. When applying a more strict definition for MASLD [26,27] and thus after excluding individuals consuming alcohol daily or being infected by HBV, the observed MASLD remained unaltered (27.8%). The 17.4% prevalence of lean SLD was higher than the according 11.2% estimate derived by a meta-analysis of European and Asian studies [28]. Heterogeneity in study design, settings (urban vs. rural), ethnic subgroups, as well as age and co-morbidity distribution between studies may account for the observed differences. Additionally, differences in imaging techniques (e.g. qualitative B-mode echography, quantitative transient electrography (TE), and magnetic resonance imaging (MRI)), may alter the precision of estimating SLD prevalence.

SLD in the absence of obesity remains poorly understood. The study setting with its high prevalence of OV infections offered the opportunity to explore for the first time in humans the potential role of these liver flukes in lean and non-lean SLD. The observed inverse association between OV infection and lean SLD is consistent with experimental evidence in fructose-fed hamsters, where OV infection decreased insulin resistance (IR) and liver fat accumulation. However, histopathologically, an increase in SLD severity was observed in this hamster model [29]. A cross-sectional study in adults from a high-prevalence OV setting in rural Thailand found a positive association between OV infection status and elevated BMI, possibly as a result of an inflammatory effect on fat deposition. Liver fat accumulation was not assessed [30].

The interplay between OV infection and MetS-related factors in SLD has not been studied in humans. Helminth infections generally improve metabolic dysfunctions to varying degrees [31]. In the case of OV, the observed inverse association with SLD could potentially be mediated by a reduced risk of MetS. The restriction of the protective association to lean persons can be explained by an infection associated weight loss with a subsequent decrease MetS risk. A case-control study in Thailand found that OV-positive individuals had slightly lower levels of HbA1c and slightly higher levels of HDL. Both parameters increased after six months of PZQ treatment [32]. In contrast, we previously reported on the absence of an association between OV infection and prediabetes or diabetes in Lao PDR [13]. Although our study focused on OV infection, in a post-hoc analysis, we also investigated the distribution of other intestinal helminth infections by SLD status. No associations were seen with hookworm infection (2,122 total cases, 76.0% in non-SLD vs 72.6% in SLD), *Trichuris trichiura* (58 total cases, 1.9% in non-SLD vs 2.5% in SLD), *Schistosoma mekongi* (33 total cases, 1.1% in non-SLD vs 1.4% in SLD), *Enterobius vermicularis* (14 total cases, 0.5% in non-SLD vs 0.4% in SLD), *Ascaris lumbricoides* (16 total cases, 05% in non-SLD vs 0.6% in SLD), and *Taenia* spp. (12 total cases, 0.4% in non-SLD vs 0.5% in SLD) (data no shown).

To further improve our understanding of the complex interrelation between helminth infections, MetS, and SLD, research into the potential mediating role of alterations in the gut microbiome is of relevance. Preliminary evidence suggests that helminth infections may protect against SLD by promoting glycolipid metabolism homeostasis, regulating inflammation, and restoring the balance of gut microbiota [31]. Metabolic dysfunction, altered gut microbiome, and dysregulated adaptive and innate immunity are cornerstones of SLD; this also applies to Asian populations [2]. OV infections are associated with gut microbiome alterations consistent with liver pathologies [2].

Similar to helminth infections, chronic hepatitis resulting from HBV or HCV has also been linked to MetS-related factors and SLD [33]. HBV has been inversely associated with the development of MetS and hepatic steatosis, whereas HCV infection has not shown the same association [34]. In a large Korean cohort, HBsAg-positive status was linked with a lower SLD risk [35]. In a Taiwanese cohort, HBsAg-positive individuals had also a lower hepatic steatosis, and an HBsAg seroclearance led to an increase in SLD [36]. The prevalence of HBsAg-positivity in the current study was insufficiently low to assess the association with SLD, but trends indicating an association with SLD were observed, which is consistent with the aforementioned studies.

BMI increased the risk of SLD and its severity in this study. Regardless of BMI status, T2DM was the factor most strongly associated with SLD. The effects sizes did not materially differ between the two groups. The same observation applied to dyslipidemia (in non-lean persons), although the effect sizes were smaller than for T2DM. It has been observed before, that MetS related factors exhibit similar associations with lean and non-lean SLD [9,37]. This may in part be explained by BMI being insufficient to adequately classify lean and non-lean SLD as it does not reflect the distribution of body fat, and in particular the presence of visceral adipose tissue, which is more relevant for MetS and IR. T2DM and other MetS-related factors are known causes of IR [38]. During IR, regardless of obesity status, peripheral cells undergo lipolysis, affecting the transportation of fatty acids to the liver and increasing hepatic lipogenesis [6,8]. The observed positive association of alcohol consumption with non-lean SLD may also be IR mediated. Alcohol consumption, in particular at a moderate level, has been found to interfere with lipid metabolism in the human liver in a manner similar to IR. It was also found to increase IR and T2DM risk [39,40]. In addition, the positive association of being wealthier with non-lean SLD is likely reflecting the more sedentary lifestyle of richer participants, which comes with a higher risk of abdominal obesity.

In Lao PDR, the adoption of modern lifestyles may increase the prevalence of SLD in the coming decades. The preference for Westernized eating habit, characterized by higher rates of eating out and consumption of fatty meat and fried/stirred-fried foods, has been observed in the capital city of Lao PDR [41]. Changes in dietary and activity habits likely underlie the trend towards increased prevalence of MetS among adults and elderly individuals in the Lao population. According to population-based surveys conducted in 2013 and 2018, respectively, T2DM prevalence increased from 8.4% among adults aged 35 years or older [42] to 22.8% in a similar-aged Lao population group [13].

The higher prevalence of SLD in both lean and non-lean women, even after adjusting for other covariates is noteworthy. A recent review points to the various female gender related aspects that have the potential to influence SLD risk over the life course, including age at menarche, age at menopause, as well as other hormone related factors [43].

## Strengths and limitations of the study

The strengths of the study include the detailed characterization of its participants for potential determinants of both, lean and non-lean SLD, and the population-based sampling in the 21 study villages. Yet, the fact that the 21 villages were purposively selected limits the generalizability of the results to the entire rural population in the two districts and to rural Lao PDR. The cross-sectional design did not allow differentiating between cause and effect or conducting mediation analysis. In the case of MetS the vicious cycle of a bidirectional relation between SLD and T2DM is well established. In the case of OV infection, it is not known whether the presence of SLD would impact on the persistence of OV infections.

Additionally, the main technique used for measuring steatotic liver disease in this study was a quantitative B-mode ultrasound, which may have lower sensitivity and specificity for detecting SLD compared to more sensitive methods such as TE, MRI, or tissue histology. This could have led to misclassification of SLD, particularly of non-severe stages, most likely resulting in the underestimation of associations.

We did not assess hepatitis C infection although it is known to be associated with both insulin resistance and SLD. According to a recent 2022 study in Lao PDR, the prevalence of HCV was reported to be low; at 1.6% [44]. Yet, we cannot exclude that some of the observed morbidity might be due to HCV infection.

The study may have been slightly underpowered to detect statistically significant effects for HBV and alcohol consumption, as potential causes of hepatic fat accumulation in SLD. Significant alcohol consumption for SLD development is typically defined as more than 21 standard drinks (one standard drink contains approximately 14 grams of pure alcohol) per week in men and more than 14 drinks per week in women over a 2-year period [26,27]. However, alcohol consumption in the current study setting did not reach these high levels, which could explain the absence of observed associations among lean persons. The assessment of alcohol consumption may have lacked the precision for assessing this level alcohol consumption, as it did not differentiate between types of alcoholic beverages consumed. Additionally, the prevalence of HBV was also too rare to estimate its association with SLD.

## Conclusion

The cross-sectional study shows a high prevalence of SLD in Laotian adults living in rural areas, affecting more females and non-lean individuals compared to males and lean group, respectively. In lean participants, an infection with OV was associated with reduced prevalence of SLD. SLD was more prevalent in individuals with T2DM, independent of BMI. The observed positive associations of metabolic syndrome components (i.e. T2DM) with SLD even among the lean is of concern, given the trend towards unhealthier diet and more sedentary lifestyles in the country. SLD comes on top of an already high burden of infection-related liver morbidity in the country. Research into the progression of SLD to severe liver morbidity and the implementation of efficient prevention and screening programs is of utmost priority.

## Data Availability

Data is available upon reasonable request to the corresponding author.

## Appendix 1. Distributions of participants among individuals who were contracted, enrolled, and included in the analysis

**Table:**

No	Village name	Targeted participants			Complete US examination			Participants used in analysis		
		N	Female, (%)	Mean age (±SD)	N	Female, (%)	Mean age (±SD)	N	Female, (%)	Mean age (±SD)
Champasack province										
1	Donesome	181	93 (51.4)	49.0 (±11.4)	60	31 (51.7)	48.4 (±10.9)	47	26 (55.3)	47.0 (±12.5)
2	Donekaden	104	59 (56.7)	51.4 (±11.5)	93	51 (54.8)	50.7 (±11.0)	85	49 (57.7)	50.2 (±10.9)
3	Donekon	553	329 (59.5)	50.3 (±11.9)	329	198 (60.2)	50.5 (±11.9)	284	172 (60.6)	49.9 (±11.5)
4	Donepheuy	77	44 (57.1)	50.2 (±12.1)	47	29 (61.7)	50.9 (±11.4)	2	2 (100.0)	43 (±9.9)
5	Kaengkoum	216	120 (55.6)	48.6 (±10.9)	137	88 (64.2)	48.8 (±10.8)	90	65 (72.2)	47.8 (±10.6)
6	Meungsaen	619	320 (51.7)	49.9 (±11.5)	322	185 (57.5)	48.1 (±9.7)	272	151 (55.5)	48.3 (±9.8)
7	Nangkaud	198	118 (59.6)	52.8 (±13.3)	105	62 (59.1)	51.3 (±11.2)	64	41 (64.1)	51.9 (±11.8)
8	Phonpeuy	241	141 (58.5)	50.4 (±12.4)	133	85 (63.9)	49.1 (±10.6)	121	82 (67.8)	48.7 (±10.5)
9	Saenhard-noi	193	101 (52.3)	50.1 (±11.9)	86	51 (59.3)	49.7 (±11.7)	41	23 (56.1)	51 (±11.4)
10	Thaphao	156	91 (58.3)	47.0 (±10.5)	98	62 (63.3)	46.3 (±10.2)	73	44 (60.3)	46.4 (±9.8)
11	Thapho-tai	228	123 (54.0)	51.8 (±12.1)	98	50 (51.0)	50.3 (±10.1)	80	41 (51.3)	48.6 (±9.5)
12	Vernsom	582	315 (54.1)	49.2 (±11.7)	338	216 (63.9)	49.8 (±10.9)	236	156 (66.0)	48.9 (±10.9)
Savannakhet province										
1	Dongmeung	258	145 (56.2)	51.9 (±12.3)	137	82 (59.9)	50.3 (±10.8)	122	75 (61.5)	50.6 (±10.8)
2	Nonkoun	193	108 (56.0)	51.6 (±12.2)	99	49 (49.05)	99 (±52.1)	72	35 (48.6)	51.7 (±10.1)
3	Paiykhong	305	170 (55.7)	49.2 (±11.2)	153	89 (58.2)	49.9 (±10.7)	131	76 (48.6)	49.5 (±10.3)
4	Phondok	234	133 (56.8)	48.3 (±10.5)	136	85 (62.5)	47.3 (±8.3)	123	78 (63.4)	46.7 (±8.0)
5	Phonmaung	665	374 (56.2)	48.9 (±10.0)	356	224 (62.9)	49.7 (±9.6)	291	190 (65.3)	49.9 (±9.7)
6	Sakeun-neu	527	292 (55.4)	51.0 (±11.5)	325	198 (60.9)	50.9 (±11.0)	234	151 (64.5)	50.2 (±11.2)
7	Sakeun-tai	401	217 (54.1)	51.5 (±12.1)	258	157 (60.8)	52.2 (±11.0)	228	138 (60.5)	52.4 (±11.4)
8	Tharmaung	243	130 (53.5)	52.6 (±23.2)	177	96 (54.2)	51.9 (±12.5)	149	81 (54.4)	51.6 (±12.5)
9	Tharmeung	276	149 (54.0)	49.9 (±10.9)	96	52 (54.2)	51.7 (±8.7)	81	46 (56.8)	51.2 (±8.9)
Total		6,450	3,572 (55.4)	50.2 (±12.2)	3,583	2,140 (59.7)	50.0 (±10.8)	2,826	1,722 (60.9)	49.7 (±10.7)

*Note.* N represents the number of participations; US: ultrasound; ±SD indicated the values as mean ± standard deviation.

## Appendix 2. Distribution of steatotic liver disease status according to participant characteristics, stratified by BMI < 23.0 kg/m^2^ versus BMI ≥23.0 kg/m^2^.

**Table:**

Covariates		Lean (*n* = 1,552)			Non-Lean (*n* = 1,274)	
		Not SLD, n (%)	SLD, n (%)		Not SLD, n (%)	SLD, n (%)
		(*n* = 1,282)	(*n* = 270)		(*n* = 767)	(*n* = 507)
Socio-demographics and economics						
Overall	–	1,282 (82.6)	270 (17.4)		767 (60.2)	507 (39.8)
Gender	male	540 (85.4)	92 (14.6)		316 (67.0)	156 (33.1)
	female	742 (80.7)	178 (19.4)		451 (56.2)	351 (43.8)
Age groups	35-49yr	677 (82.5)	144 (17.5)		435 (62.2)	264 (37.8)
	50-59yr	334 (80.3)	82 (19.7)		185 (54.3)	156 (45.8)
	≥60yr	271 (86.0)	44 (14.0)		147 (62.8)	87 (37.2)
Education	illiterate	250 (83.1)	51 (16.9)		97 (53.6)	84 (46.4)
	primary school	584 (79.7)	149 (20.3)		361 (60.6)	235 (39.4)
	above secondary school	448 (86.5)	70 (13.5)		309 (62.2)	188 (37.8)
Profession	housewife/elderly	111 (87.4)	16 (12.6)		68 (57.1)	51 (42.9)
	farmer/labour	1,037 (83.6)	204 (16.4)		601 (61.2)	381 (38.8)
	civil servants/traders	134 (72.8)	50 (27.2)		98 (56.7)	75 (43.4)
SES	poor tertile	474 (87.3)	69 (12.7)		315 (62.3)	191 (37.8)
	middle tertile	399 (80.8)	95 (19.2)		274 (65.7)	143 (34.3)
	wealthy tertile	409 (79.4)	106 (20.6)		178 (50.7)	173 (49.3)
Province	CPS	620 (75.1)	206 (24.9)		327 (57.5)	242 (42.5)
	SVK	662 (91.2)	64 (8.8)		440 (62.4)	265 (37.6)
Lifestyle						
Smoking	non-smoker	815 (83.4)	162 (16.6)		525 (58.9)	366 (41.1)
	smoker, current & previous	467 (81.2)	108 (18.8)		242 (63.2)	141 (36.8)
Alcohol	less	1,229 (82.6)	259 (17.4)		748 (60.4)	491 (39.6)
	daily	53 (82.8)	11 (17.2)		19 (54.3)	16 (45.7)
Healthy diet	yes	1,208 (82.3)	260 (17.7)		732 (60.6)	476 (39.4)
	no	74 (88.1)	10 (11.9)		35 (53.0)	31 (47.0)
Health risks						
T2DM	not T2DM	1,213 (84.2)	227 (15.8)		709 (64.0)	398 (36.0)
	T2DM	69 (61.6)	43 (38.4)		58 (34.7)	109 (65.3)
BP	normal BP	1,073 (83.5)	212 (16.5)		580 (62.8)	343 (37.2)
	increased BP	209 (78.3)	58 (21.7)		187 (53.3)	164 (46.7)
Lipids	normal	132 (86.3)	21 (13.7)		62 (72.9)	23 (27.1)
	dyslipidemia	1,150 (82.2)	249 (17.8)		705 (59.3)	484 (40.7)
OV infection	OV neg	127 (69.4)	56 (30.6)		100 (56.5)	77 (43.5)
	OV pos	1,155 (84.4)	214 (15.6)		667 (60.8)	430 (39.2)
HBV infection	HBV neg	1,243 (82.4)	266 (17.6)		736 (59.8)	495 (40.2)
	HBV pos	39 (90.7)	4 (9.3)		31 (72.1)	12 (27.9)

*Notes.* SLD: steatotic liver disease; n (%): the number of participants (percentage); yr: year; SES: social-economic status; CPS: Champasak; SVK: Savannakhet; T2DM: type 2 diabetes mellitus; BP: blood pressure; OV: *Opisthorchis viverrini*; neg: negative; pos: positive; HBV: hepatitis B virus.

## Appendix 3. Associations between covariates and steatotic liver disease, stratified by body mass index (lean < 23kg/m^2^ and not lean ≥23 kg/m^2^).

**Table:**

Covariates		Lean		Non-lean	
		Univariate	Fully adjusted	Univariate	Fully adjusted
		cOR (95% CI)	aOR (95% CI)	cOR (95% CI)	aOR (95% CI)
OV infection	OV neg	ref	ref	ref	ref
	OV pos	0.53 (0.37 − 0.77)**	0.49 (0.33 − 0.73)***	0.87 (0.62 − 1.22)	0.93 (0.65 − 1.32)
HBV infection	HBV neg	ref	ref	ref	ref
	HBV pos	0.46 (0.16 − 1.33)	0.42 (0.14 − 1.25)	0.55 (0.28 − 1.08)	0.65 (0.32 − 1.31)
T2DM	non DM	ref	ref	ref	ref
	T2DM	3.54 (2.23 − 5.50)***	3.58 (2.28 − 5.63)***	3.41 (2.41 − 4.83)***	3.31 (2.31 − 4.74)***
Lipids	normal	ref	ref	ref	ref
	dyslipidemia	1.36 (0.83 − 2.24)	1.39 (0.83 − 2.32)	1.91 (1.15 − 3.15)*	1.83 (1.09 − 3.07)*
Smoking	non-smoker	ref	ref	ref	ref
	smoker, current & prev	1.08 (0.80 − 1.44)	1.33 (0.94 − 1.88)	0.77 (0.59 − 1.00)	0.85 (0.63 − 1.15)
Alcohol	less	ref	ref	ref	ref
	daily	0.86 (0.43 − 1.72)	1.07 (0.52 − 2.20)	1.28 (0.63 − 2.58)	2.08 (0.98 − 4.42)
Healthy diet	yes	ref	ref	ref	ref
	no	0.74 (0.37 − 1.48)	0.69 (0.34 − 1.41)	1.37 (0.83 − 2.27)	1.29 (0.76 − 2.19)
Gender	male	ref	ref	ref	ref
	female	1.41 (1.06 − 1.87)*	1.43 (1.02 − 2.01)*	1.59 (1.25 − 2.02)***	1.50 (1.12 − 2.01)**
Age groups	35-49yr	ref	ref	ref	ref
	50-59yr	1.19 (0.87 − 1.63)	0.94 (0.68 − 1.32)	1.42 (1.09 − 1.86)*	1.30 (0.98 − 1.73)
	≥60yr	0.82 (0.56 − 1.20)	0.68 (0.44 − 1.05)	0.99 (0.72 − 1.35)	0.83 (0.58 − 1.19)
Education	illiterate	ref	ref	ref	ref
	primary school	0.90 (0.62 − 1.31)	0.82 (0.55 − 1.23)	0.76 (0.54 − 1.07)	0.78 (0.54 − 1.14)
	above secondary	0.60 (0.40 − 0.91)*	0.53 (0.33 − 0.85)**	0.71 (0.50 − 1.01)	0.80 (0.53 − 1.20)
Profession	housewife/elderly	ref	ref	ref	ref
	farmer/labour	1.45 (0.82 − 2.55)	1.50 (0.82 − 2.73)	0.88 (0.59 − 1.31)	0.96 (0.63 − 1.46)
	civil servants/traders	1.59 (0.80 − 3.17)	1.80 (0.85 − 3.82)	1.07 (0.66 − 1.75)	1.17 (0.68 − 2.00)
SES	poor tertile	ref	ref	ref	ref
	middle tertile	0.0.93 (0.62 − 1.41)	0.95 (0.62 − 1.45)	0.87 (0.65 − 1.16)	0.88 (0.65 − 1.19)
	wealthy tertile	1.06 (0.61 − 1.85)	1.14 (0.65 − 2.00)	1.73 (1.21 − 2.47)**	1.72 (1.17 − 2.53)**

*Note.* OV: *Opisthorchis viverrini*; neg: negative; pos: positive; HBV: hepatitis B virus; T2DM: type 2 diabetes mellitus; smoking prev: smoking previously; yr: year; SES: social-economic status; cOR: crude odds ratio; aOR: adjusted odds ratio; CI: confidence interval.*p-value < 0.05, **p-value < 0.01, ***p-value < 0.001.

## Appendix 4. Associations between covariates and steatotic liver disease according to sensitivity analysis: (a) excluding participants reporting daily alcohol consumption and/or tested positive for hepatitis B surface antigen, and (b) stratified by BMI <25.0 kg/m^2^ (lean) versus BMI ≥25.0 kg/m^2^ (non-lean)

**Table:**

Covariates		Excluded daily alcohol and HBV pos		BMI cut-off at 25 kg/m^2^	
		Lean	Non-lean	<25 kg/m^2^	≥25 kg/m^2^
		aOR (95% CI)	aOR (95% CI)	aOR (95% CI)	aOR (95% CI)
OV infection	OV neg	ref	ref	ref	ref
	OV pos	0.50 (0.33 − 0.74)***	0.89 (0.62 − 1.29)	0.60 (0.44 − 0.83)**	0.94 (0.59 − 1.49)
HBV infection	HBV neg	–	–	ref	ref
	HBV pos	–	–	0.46 (0.20 − 1.04)	0.84 (0.34 − 2.10)
DM	non T2DM	ref	ref	ref	ref
	T2DM	3.67 (2.33 − 5.78)***	3.46 (2.39 − 5.01)***	3.75 (2.63 − 5.34)***	3.09 (1.95 − 4.90)***
Lipids	normal	ref	ref	ref	ref
	dyslipidemia	1.42 (0.83 − 2.44)	2.12 (1.20 − 3.73)**	1.61 (1.04 − 2.49)*	1.62 (0.83 − 3.17)
Smoking	non-smoker	ref	ref	ref	ref
	smoker, current & previous	1.36 (0.83 − 2.44)	0.86 (0.63 − 1.18)	1.16 (0.88 − 1.53)	0.75 (0.50 − 1.13)
Alcohol	less	–	–	ref	ref
	daily	–	–	1.08 (0.58 − 2.01)	2.87 (1.05 − 7.89)*
Healthy diet	yes	ref	ref	ref	ref
	no	0.72 (0.35 − 1.48)	1.37 (0.79 − 2.36)	0.76 (0.43 − 1.35)	1.21 (0.64 − 2.30)
Gender	male	ref	ref	ref	ref
	female	1.50 (1.06 − 2.13)*	1.51 (1.12 − 2.04)**	1.36 (1.03 − 1.79)*	1.45 (1.00 − 2.13)
Age groups	35-49yr	ref	ref	ref	ref
	50-59yr	0.91 (0.65 − 1.29)	1.33 (0.99 − 1.77)	1.11 (0.85 − 1.45)	1.14 (0.79 − 1.65)
	≥60yr	0.64 (0.41 − 1.00)	0.88 (0.61 − 1.28)	0.75 (0.53 − 1.05)	0.79 (0.50 − 1.26)
Education	illiterate	ref	ref	ref	ref
	primary school	0.81 (0.54 − 1.24)	0.77 (0.53 − 1.14)	0.86 (0.62 − 1.20)	0.78 (0.48 − 1.28)
	above secondary school	0.55 (0.34 − 0.88)*	0.80 (0.53 − 1.23)	0.64 (0.43 − 0.93)*	0.79 (0.47 − 1.34)
Profession	housewife/elderly	ref	ref	ref	ref
	farmer/labour	1.47 (0.79 − 2.74)	1.00 (0.65 − 1.55)	1.16 (0.75 − 1.79)	1.01 (0.58 − 1.77)
	civil servants/traders	1.73 (0.80 − 3.75)	1.20 (0.68 − 2.09)	1.39 (0.80 − 2.42)	1.19 (0.57 − 2.45)
SES	poor tertile	ref	ref	ref	ref
	middle tertile	0.95 (0.62 − 1.45)	0.85 (0.62 − 1.17)	0.84 (0.60 − 1.18)	0.93 (0.62 − 1.40)
	wealthy tertile	1.24 (0.71 − 2.14)	1.64 (1.08 − 2.48)*	1.06 (0.68 − 1.66)	2.11 (1.19 − 3.73)*

*Note.* OV: *Opisthorchis viverrini*; neg: negative; pos: positive; HBV: hepatitis B virus; T2DM: type 2 diabetes mellitus; smoking prev: smoking previously; yr: year; SES: social-economic status; aOR: adjusted odds ratio; CI: confidence interval.*p-value < 0.05, **p-value < 0.01, ***p-value < 0.001.
